# Magnetic Resonance Imaging Findings and Potential Anatomic Risk Factors for Anterolateral Ankle Impingement in Children and Adolescents Suffering from Non-Overload Atraumatic Ankle Pain

**DOI:** 10.3390/diagnostics14202265

**Published:** 2024-10-11

**Authors:** Wolf Bäumler, Josina Straub, Johannes Weber, Patrick Ostheim, Julia Lenz, Volker Alt, Christian Stroszczynski, Jan Reinhard, Daniel Popp

**Affiliations:** 1 Department of Radiology, University Hospital Regensburg, 93053 Regensburg, Germany; christian.stros@ukr.de; 2 Department of Trauma Surgery, University Hospital Regensburg, 93053 Regensburg, Germany; josina.straub@ukr.de (J.S.); johannes1.weber@ukr.de (J.W.); volker.alt@ukr.de (V.A.); daniel.popp@ukr.de (D.P.); 3 Bundeswehr Institute of Radiobiology, Affiliated to the University of Ulm, 80937 Munich, Germany; patrickostheim@bundeswehr.org; 4 Department of Orthopedic Surgery, University Hospital Regensburg, 93077 Bad Abbach, Germany; jan.reinhard@ukr.de

**Keywords:** magnetic resonance imaging (MRI), ankle pain, atraumatic, non-overload, children, adolescents, anterolateral impingement, risk factors, hindfoot alignment, hindfoot valgus

## Abstract

Background/Objectives: To assess magnetic resonance image (MRI) findings in children and adolescents with atraumatic non-overload ankle pain and to identify potential anatomic risk factors. Methods: In total, 310 MRIs of 6- to 20-year-old patients were evaluated regarding detectable ankle pathologies. A total of 147 patients (68 males; 79 females) suffered from atraumatic non-overload ankle pain. The findings were compared to a control group (163 patients: 89 males; 74 females), including patients with ankle trauma in the 4 weeks prior to MRI examination. A *t*-test for unpaired samples and a binary logistic regression model were used to identify significant differences between both groups and determine potential anatomic risk factors. Results: In the group with atraumatic ankle pain, 95 patients (64.6%) showed at least one pathology. Anterolateral impingement of the upper ankle joint was found in 29 patients (19.7%). Its occurrence was significantly higher in atraumatic non-overload patients than in the control group (*p* = 0.043). Moreover, a significant correlation between anterolateral impingement of the upper ankle and the presence of hindfoot valgus malposition (*n* = 25; 17.0%) could be proven in atraumatic non-overload patients (*p* = 0.035). Conclusions: Anterolateral impingement of the upper ankle joint is frequently observed in children and adolescents suffering from atraumatic non-overload ankle pain, whereby a hindfoot valgus malposition seems to present an anatomic risk factor.

## 1. Introduction

Ankle pain is often seen in younger patients and adolescents. It can be caused by anterolateral impingement of the upper ankle, which ranks among the most frequent pathologies of this joint and is typically seen in younger athletes [1,2]. As well as in young athletes, who are frequently exposed to physical overload, anterolateral impingement can also be observed in young patients without a history of physical overload or trauma. Anterolateral impingement resembles intra-articular soft tissue entrapment or even osseous impingement at the anterolateral distal tibia [3]. This alteration leads to pain and a reduced range of motion in the tibiotalar joint [4]. It is assumed that minor injuries caused by forced plantar flexion and inversion lead to micro-instability, resulting in the thickening of synovia and scarring [5]. The abnormally increased tissue in the anterolateral aspect of the joint might restrict the dorsiflexion of the talus and lead to painful entrapment [2]. In terms of osseous impingement, a spur formation along the anterior margin of the distal tibia and talus is observed.

Diagnosis is made upon clinical examination and with the help of radiological imaging techniques. Regarding radiological diagnostics, a conventional X-ray is usually performed, as it helps to diagnose osseous factors contributing to impingement reliably and represents a cost-effective and comprehensively applied diagnostic tool. Although it may show ossicles, the conventional X-ray plays a minor role in the diagnosis of anterolateral impingement of the ankle if all its potential causes are considered, whereas Arthro-computed tomography (CT) is able to indicate anterolateral impingement [3]. Ultrasound is also suitable but has been shown to be inferior in diagnosis in comparison to Arthro-CT [6]. Conventional magnetic resonance imaging (MRI) represents the gold standard and shows a very high accuracy in detecting abnormally increased tissue in the anterolateral recess [4,7]. A large number of publications discuss anterolateral impingement of the ankle, for example, comparing its operative and non-operative treatment [8,9,10,11]. However, most of these trials include athletes, who are often exposed to physical overload. In the current literature, there is hardly any information about anterolateral impingement or ankle pain in young patients without a history of traumata or physical overload. Therefore, in this retrospective analysis, we aimed to investigate MRI findings and determine potential influencing factors in children and adolescents suffering from non-overload atraumatic ankle pain.

## 2. Material and Methods

### 2.1. Study Design, Participant Selection and Patient Characteristics

A retrospective study design was chosen to conduct this study. The trial was performed according to the current guidelines and regulations and was approved by the Ethics Committee of the University of Regensburg (approval number: 24-3625-104; date: 25 January 2024). The authors evaluated all magnetic resonance tomographies of the ankle performed between 1 February 2021 and 30 April 2024 in patients between 6 and 20 years of age. MRIs were performed in a radiological practice. We aimed to investigate MRI findings in order to identify potential influencing factors of young patients suffering from non-overload atraumatic ankle pain.

The inclusion criteria were defined as follows: (I) An orthopedic examination of the affected ankle conducted within the last quarter of the year. The examination had to be performed by an orthopedist. (II) A referral for an MRI by an orthopedist. (III) Absence of physical overload history or trauma of the ankle. All information was discussed and documented in a detailed anamnesis interview performed by the orthopedist. Furthermore, this information was requested by the radiologist again to avoid incorrect inclusion. (IV) No history of surgery on the affected ankle. This information was also discussed in the orthopedic and radiological anamnesis interview. (V) The patient signed an informed consent for the MRI examination and the anonymous use of the acquired data for scientific publication.

To create the control group, magnetic resonance tomographies of children and adolescents of the same age with a traumatic injury of the ankle during the 4 weeks prior to examination were analyzed. Moreover, the mentioned inclusion criteria, IV and V, had to be fulfilled.

In total, the magnetic resonance tomographies of 310 patients were evaluated. Of these, 147 patients were included in the atraumatic non-overload ankle pain group: 68 males (46.3%) and 79 females (53.7%). Mean age in the atraumatic ankle pain group was 15.8 ± 3.3 years (range: 6–20 years). In 72 cases (49.0%), the right ankle was affected, while 75 patients (51.0%) suffered from left ankle pain.

The control group consisted of 163 patients (89 males, 54.6%; 74 females, 45.5%). Mean age was 16.4 ± 3.1 years (range: 6–20 years). A total of 90 patients (55.2%) had experienced trauma of the right ankle; in 73 cases (44.8%) the left ankle was affected.

### 2.2. Image Acquisition and Evaluation

To acquire the data, a 1.5 Tesla MR scanner (MAGNETOM Altea, Siemens Healthcare GmbH, Erlangen, Germany) and a picture archiving and communication system (PACS) were used. To ensure standardized examinations, a standardized testing protocol was applied, including sagittal fat-saturated proton density-weighted turbo spin echo (PD-tse-fs) images [repetition time (TR)/echo time (TE) 3000/32 ms; 320 × 75 matrix; flip angle: 150°; thickness: 2 mm]. Furthermore, it contained coronal T1-tse (TR/TE 752/10 ms; 256 × 95 matrix; flip angle: 180°; thickness: 2 mm), coronal PD-tse-fs (TR/TE 3000/33 ms; 256 × 95 matrix; flip angle: 150°; thickness: 2 mm) and axial PD-tse-fs (TR/TE 3300/38 ms; 320 × 70 matrix; flip angle: 150°; thickness: 3 mm) images. The standardized field of view (FOV) was set to 160 mm. The technical parameters of the MRI protocol are summarized in Table 1.

The MRI images were evaluated independently by two radiologists with 9 years and 10 years of experience. Both observers are certified in musculoskeletal imaging. After the individual analysis, the results were compared. In cases of discrepancy, the case was discussed in detail, and a consensus decision was made. The radiologists evaluated the upper ankle joint, the subtalar joint, the Chopart articulations and the flexor and extensor mechanism of the ankle.

**Figure 1:**
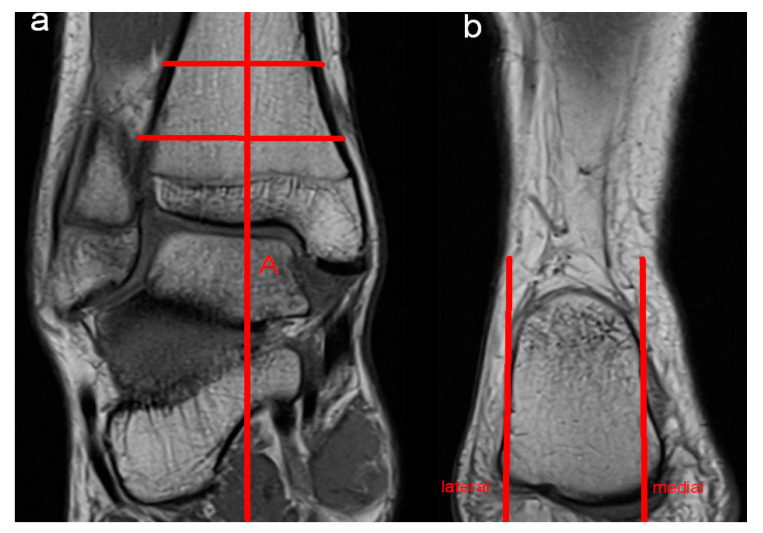
Hindfoot valgus was evaluated by measuring the angle between the tibial shaft axis (A) (**a**) and a line adapted to the medial and lateral surfaces of the calcaneus (**b**).

**Figure 2:**
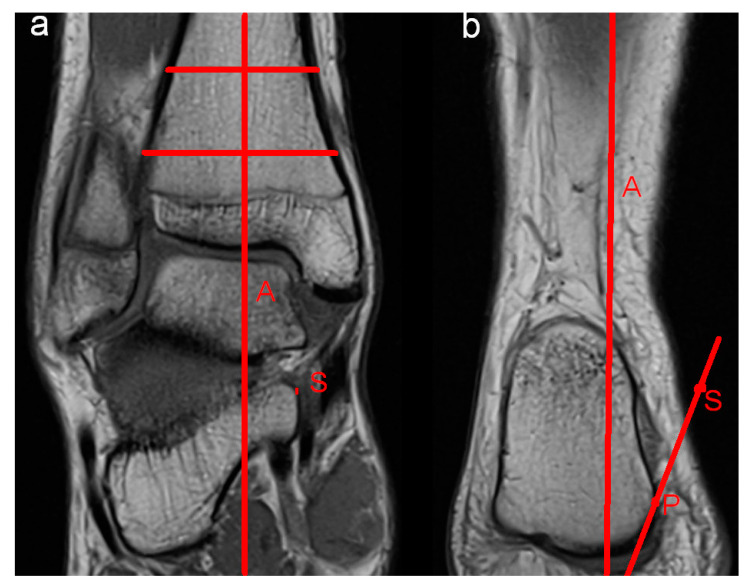
Hindfoot varus was evaluated by measuring the angle between the tibial shaft axis (A) (**a**) and a line drawn at a tangent from the tip of the sustentaculum tali (S) to the plantar medial surface (P) of the calcaneus (**b**).

**Figure 3:**
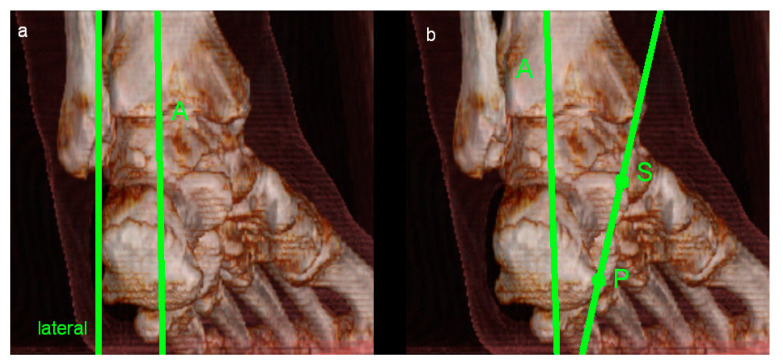
Illustration of the measurement methods of hindfoot valgus (**a**) and hindfoot varus (**b**) in a 3-dimensional model.

Moreover, potential anatomic risk factors for the development of perspective ankle complaints were assessed. MRI evaluation included abnormalities of the bone, the ventral and dorsal syndesmosis, the collateral ligaments, ligamentum bifurcatum, cartilage (modified Outerbridge classification; Grad I: cartilage with focal inhomogeneities but without substantial defect; Grade II: focal defect up to 50% of the cartilage height; Grade III: focal damage > 50% of the cartilage height without total height reduction; Grade IV: presence of an area with complete height reduction in cartilage), Osgood Schlatter disease, articular effusion, tarsal coalition, flexor and extensor mechanisms of the ankle (rupture or partial rupture of muscles or tendons, tendinopathy), bursa subachillae, plantar fascia and other intra-articular abnormalities, such as the presence of intra-articular loose bodies. Furthermore, impingement (anterolateral, anterior, medial and posterior) of the ankle was assessed. Impingement was diagnosed if the distance of the joint cavity was measured < 2 mm in the respective part of the joint. A reduction in the joint cavity could be caused by soft tissue or by the articulating surfaces themselves. To diagnose anterolateral impingement, the anterior joint cavity between the distal fibula and the distal tibia was evaluated by axial and coronal imaging. Sagittal and axial imaging was used to assess anterior impingement between the anterior part of the distal tibia and the talus. Coronal and axial imaging was evaluated to diagnose medial impingement between the malleolus medialis and the medial shoulder of the talus. Sagittal and axial imaging was used to assess posterior impingement between the dorsal part of the distal tibia and the processus posterior tali and/or the calcaneus. Moreover, hindfoot alignment was evaluated radiologically by coronal imaging. Referring to the findings of Buck et al., hindfoot valgus was assessed by the angle between the tibial shaft axis and a line adapted to the medial and lateral surfaces of the calcaneus. An angle > 11° was defined as hindfoot valgus malposition. Hindfoot varus was evaluated by the angle between the tibial shaft axis and a line drawn at a tangent from the tip of the sustentaculum tali to the plantar medial surface of the calcaneus. Values < 12° were defined as hindfoot varus malposition [12]. Figure 1, Figure 2 and Figure 3 illustrate the mentioned measurement methods.

### 2.3. Statistical Analysis

All collected data are presented as frequency counts and percentages. To evaluate potential differences in the prevalence of several variants of ankle impingement between the two patient groups, a *t*-test for unpaired samples was applied. Furthermore, binary logistic regression models were utilized to identify potential influencing factors on the different variants of upper ankle impingement and to determine a potential correlation between articular effusion and hindfoot malalignment or ankle impingement. It was demonstrated that 95% confidence intervals were used as effect estimates. According to common international practice, a *p*-value of ≤0.05 was defined as being statistically significant. To perform a statistical analysis, SPSS statistics (IBM SPSS Statistics, version 28, IBM, Armonk, NY, USA) was used.

## 3. Results

For all data, a normal distribution could be proven. In 95 of the 147 patients (64.6%) in the atraumatic ankle pain group, at least one pathology was detected. The most common pathology was articular effusion in the upper ankle joint (*n* = 32; 21.8%). Similar to the articular effusion in the subtalar joint, it was mostly associated with another pathology (chondral lesion in the upper ankle joint: *n* = 4; osteochondritis dissecans in the upper ankle joint: *n* = 1; anterolateral impingement: *n* = 8; anterior impingement: *n* = 1; medial impingement: *n* = 2; posterior impingement: *n* = 2, rupture of the M. peroneus longus tendon: *n* = 1; tendinopathy of the M. tibialis posterior tendon: *n* = 2; tendinopathy of the M. flexor digitorum longus tendon: *n* = 1; hindfoot valgus: *n* = 7; hindfoot varus: *n* = 1). Only in two cases (1.4%) was articular effusion observed as a solitary finding in both joints. The second most frequently detected finding was anterolateral impingement of the upper ankle joint (*n* = 29; 19.7%), followed by articular effusion in the subtalar joint (*n* = 17; 11.6%) and tendinopathy of the musculus tibialis posterior tendon (*n* = 13; 8.8%). In the control group, each patient was diagnosed with at least one pathology. Articular effusion of the upper ankle joint represented the most common disease (*n* = 71; 43.6%), always being associated with another detectable pathology. The second and third most commonly observed diseases in the control group were ruptures or partial ruptures of the ligamentum fibulotalare anterius (*n* = 41; 25.2%) and bone edema (*n* = 35; 21.5%). Regarding hindfoot alignment, a valgus malposition could be proven in 25 (17.0%) atraumatic non-overload patients and in 5 (3.1%) trauma patients. A varus malposition was detected in 5 (3.4%) patients without traumatic or overload history and in 3 (1.8%) cases of the control group. All pathological findings are summarized in Table 2. Comparing the occurrence of the several variants of upper ankle impingement in the two patient groups, anterolateral impingement was observed significantly more often in atraumatic non-overload patients than in the control group (*p* = 0.043). Regarding anterior, medial and posterior impingement, no significant differences were found. The results are presented in Table 3. As upper ankle impingement represented a frequent pathology, the potential influence of hindfoot valgus and varus on several variants of upper ankle impingement was assessed in both groups. In patients suffering from atraumatic non-overload ankle pain, a significant correlation between the occurrence of anterolateral upper ankle impingement and the presence of hindfoot valgus malposition could be proven (*p* = 0.035), as shown in Table 4. In contrast, in the control group, no significant correlation between upper ankle impingement and hindfoot valgus or varus malposition was found, as shown in Table 5. Moreover, a potential correlation between articular effusion in the upper ankle joint and hindfoot malalignment or ankle impingement in atraumatic non-overload patients was evaluated. Neither hindfoot malalignment nor ankle impingement showed a significant correlation with the presence of joint effusion. Table 6 summarizes the results.

## 4. Discussion

In this retrospective study, in 64.6% of patients (95/147) with atraumatic ankle pain, at least one pathology was detected by MRI. Most frequently, articular effusion in the upper ankle joint was found (*n* = 32; 21.8%). In almost every case, this finding was associated with another ankle pathology (*n* = 30; 20.4%). Subsequently, it can be interpreted as a consequence of the underlying disease. The second most common diagnosis was anterolateral impingement of the upper ankle joint (*n* = 29; 19.7%). Its occurrence was significantly higher in atraumatic non-overload patients than in the control group (*p* = 0.043). Moreover, a significant correlation between anterolateral impingement of the upper ankle and the presence of hindfoot valgus malposition (*n* = 25; 17.0%) could be proven in atraumatic non-overload patients (*p* = 0.035). A potential reason for this correlation might be the reduction in the lateral part of the joint space in the upper ankle being caused by valgus malalignment. This alone increases the risk of a consecutive impingement. Another possible explanation could be the fact that the anatomic malposition might affect unequal load distribution in the lateral part of the joint space, resulting in micro-traumata, which, initially, are not necessarily painful for the patient. The micro-traumata may cause thickening of the soft tissue and scarring [5], potentially leading to entrapment [2], which can be proven by MRI. The anatomical situation of the upper ankle joint, with several soft tissue components adjoining its anterolateral part, might be a reason for the frequent occurrence of impingement in the anterior part of the lateral joint space. Finally, the exact pathomechanism is hard to prove; therefore, a combination of the mentioned facts must be considered.

To the best of our knowledge, this study is the first to prove a significant correlation between anterolateral impingement of the upper ankle and the presence of hindfoot valgus malposition in young, atraumatic non-overload patients.

During the last ten years, Single Photon Emission Computed Tomography/Computed Tomography (SPECT/CT) has increasingly been used as a diagnostic tool for ankle pain [8,13]. SPECT/CT enables the illustration of focally increased bone metabolism. Therefore, different studies report its favorable use in cases of arthrosis, osteochondral lesions, impingement syndromes, coalitions and accessory bones but also in tendon and ligament pathologies of the ankle [8,14,15]. Although the authors state that this method involves minimal radiation exposure, for our younger patient cohort, this diagnostic instrument is not suitable [8]. MRI constitutes a powerful and valuable imaging tool, featuring a high image signal and excellent soft tissue contrast [16,17]. It enables the visualization of a wide range of pathologies around the ankle, which affect the articular cartilage, bone marrow, ligaments, tendons, synovium and nerves [16,18]. The advantages of the use of MRI, with its lack of radiation, are particularly emphasized in younger patients and it consequently represents the gold standard in terms of imaging techniques [17]. Especially in our special patient cohort, which consists of children and adolescents suffering from atraumatic non-overload ankle pain, high-resolution soft tissue visualization is needed to detect the smallest lesions and to enable targeted therapy [17,19].

The MR images were evaluated independently by two very experienced radiologists who are certified in musculoskeletal imaging. Each pathology, especially the anterolateral impingement, was evaluated in the PD-tse-fs sequence in all three planes (sagittal, axial, coronal). In contrast, most existing studies on anterolateral impingement of the ankle only evaluated one plane. Regarding the existing literature, most studies recommend an axial MR plane for the diagnosis of anterolateral impingement of the ankle [3]. Another study recommends using sagittal plane T1 and short tau inversion recovery (STIR) sequences for diagnosis [20]. From our point of view, a 3-dimensional assessment of the ankle, taking into account all three MRI planes, can increase the probability of identifying pathologies, especially anterolateral impingement. However, further studies are needed to determine the best MRI sequences to assess this pathology [3,20].

In a daily clinical routine, hindfoot alignment is usually assessed radiographically by using the hindfoot alignment view, also known as the Salzmann view [21]. A more recent imaging technique is the weight-bearing computed tomography (CT) of the ankle [22]. Both the hindfoot alignment view radiographs and the weight-bearing CT were proven to be highly reliable and highly correlated imaging techniques to assess hindfoot alignment [23]. The value of MRI in terms of hindfoot alignment is controversially discussed [24]. Haldar et al. could prove a significant correlation between the tibiocalcaneal angle of the ankle, using non-weight-bearing MRI and the hindfoot alignment assessed by weight-bearing CT [25]. Buck et al. discussed the possibility of assessing the hindfoot alignment using coronal non-weight-bearing MR images [12]. In the present study, we evaluated the hindfoot alignment by referring to these findings. Plain X-rays have a limited ability to detect an additional existing anterolateral impingement. Thus, the MRI represents the most suitable imaging technique to assess pathologies around the ankle in children and adolescents suffering from non-overload atraumatic ankle pain. Furthermore, MRI not only represents an imaging technique that offers a high-quality assessment of several types of tissue but also helps to identify ankle pathologies. Its data allow the creation of 3-dimensional models, which result in an extremely precise illustration of an individual anatomical situation or a present disease and may additionally improve the exact diagnosis of ankle pathologies like anterolateral impingement. Furthermore, the 3-dimensional data can be helpful in subsequent therapies. In addition to the optimized planning of surgeries because of the precise preoperative 3-dimensional illustration, the data can also be used for 3-dimensional printers, which are even able to create individual implants [26,27] if necessary. The use of 3-dimensional printed implants has especially proven its worth in difficult-to-treat cases [28]. Therefore, allied to its diagnostic strength, the use of MRI may provide additional opportunities to improve future therapies.

As well as its precise illustration of soft tissue pathologies of the ankle [4,7], MRI also offers the possibility to diagnose hindfoot alignment [12]. However, after critical reflection of radiological reports drawn up after an MR tomography of the ankle, we must admit that, in most cases, there is no information concerning the presence of hindfoot malposition. Moreover, not every observer pays enough attention to the potential presence of ankle impingement. With this MRI study indicating a correlation between anterolateral impingement of the upper ankle and the presence of hindfoot valgus malposition in young patients suffering from non-overload atraumatic ankle pain, the huge diagnostic value of MRI in ankle disease seems to increase even more. Regarding the results of the current trial, it seems to be indispensable to include an evaluation of potential ankle impingement and hindfoot alignment in the radiological report. If this information is not included as standard, important diagnostic information could be lost, possibly leading to unnecessary follow-up examinations. Consequently, this information should be integrated into the standardized radiological report systems, which are often used nowadays, especially if the patients are young and suffer from non-overload atraumatic ankle pain. A possible limitation of this study may be the use of a 1.5 Tesla MRI instead of a 3 Tesla MRI. Different studies have shown an improved diagnostic performance in 3 Tesla MRIs in assessing cartilage and ligament pathologies of the ankle [29,30,31]. However, we detected a pathology in more than half of the patients, highlighting the high diagnostic value of 1.5 Tesla MRIs. It could well be that the wider use of 3 Tesla MRIs may lead to additional diagnoses. Another limitation of the present study is the small study population. Although this study represents a rather large population regarding the existing literature, further studies are needed featuring a larger study population.

## 5. Conclusions

In this study, we established that anterolateral impingement of the upper ankle represents a frequent pathology in children and adolescents suffering from non-overload atraumatic ankle pain. The use of MRI is highly recommended in these patients, as it can offer decisive added value in diagnostics and enable a targeted therapy. Especially in children and adolescents, the hindfoot alignment assessment should be standardized, as anterolateral impingement is often associated with valgus hindfoot malalignment. This would increase awareness of this pathology, benefiting all patients.

## Figures and Tables

**Table 1:** Technical parameters of the MRI protocol.

MRI	TR/TE	Matrix	Flip Angle	Thickness	FOV
Sagittal PD-tse-fs	3000/32 ms	320 × 75	150°	2 mm	160 mm
Coronal T1-tse	752/10 ms	256 × 95	180°	2 mm	160 mm
Coronal PD-tse-fs	3000/33 ms	256 × 95	150°	2 mm	160 mm
Axial PD-tse-fs	3300/38 ms	320 × 70	150°	3 mm	160 mm

**Table 2:** Pathological findings in patients with atraumatic non-overload ankle pain and the control group.

Pathological Findings	Atraumatic Ankle Pain Group		Control Group	
	Number of Patients	%	Number of Patients	%
Bone edema	2	1.4	35	21.5
Fracture	0	0	3	1.8
Ligament rupture or partial rupture				
Lig. fibulotalare anterius	0	0	41	25.2
Lig. fibulocalcaneare	0	0	21	12.9
Lig. fibulotalare posterius	0	0	3	1.8
Lig. deltoideum	0	0	25	15.3
Lig. bifurcatum	0	0	17	10.4
Rupture of the syndesmosis	0	0	8	4.9
Chondral lesion in the upper ankle joint				
Grade I	6	4.1	7	4.3
Grade II	2	1.4	6	3.7
Grade III	0	0	2	1.2
Grade IV	0	0	0	0
Osteochondritis dissecans in the upper ankle joint	4	2.7	2	1.2
Articular effusion in the upper ankle joint	32	21.8	71	43.6
Articular effusion in the subtalar joint	17	11.6	31	19.0
Tarsal coalition	1	0.7	0	
Tendinopathy				
M. tibialis anterior	2	1.4	4	2.5
M. extensor hallucis longus	2	1.4	3	1.8
M. extensor digitorum	1	0.7	1	0.6
M. tibialis posterior	13	8.8	17	10.4
M. flexor digitorum longus	7	4.8	14	8.6
M. flexor hallucis longus	1	0.7	3	1.8
M. peroneus longus	8	5.4	9	5.5
M. peroneus brevis	6	4.1	11	6.7
Achilles tendon	5	3.4	2	1.2
Rupture of the M. peroneus longus tendon	1	0.7	0	0
Bursitis subachillae	4	2.7	2	1.2
Plantar fasciitis	2	1.4	1	0.6
Impingement				
Anterolateral	29	19.7	6	3.7
Anterior	4	2.7	2	1.2
Medial	8	5.4	5	3.1
Posterior	3	2.0	4	2.5
Hindfoot valgus	25	17.0	5	3.1
Hindfoot varus	5	3.4	3	1.8
Intra-articular loose body	0	0	2	1.2

**Table 3:** Results of a *t*-test for unpaired samples comparing several variants of ankle impingement in atraumatic non-overload patients and the control group.

Variable	*p*-Value	95% Confidence Interval
Anterolateral impingement	0.043	2.802–6.3412
Anterior impingement	0.794	−3.649–4.381
Medial impingement	0.682	−2.905–3.654
Posterior impingement	0.903	−5.043–4.942

**Table 4:** Results of the binary logistic regression model predicting the influence of hindfoot varus and hindfoot valgus on several variants of ankle impingement in atraumatic non-overload patients.

	Hindfoot Valgus		Hindfoot Varus	
Impingement	*p*-Value	95% Confidence Interval	*p*-Value	95% Confidence Interval
Anterolateral	0.035	2.132–4.325	0.641	−2.445–3.012
Anterior	0.814	−2.952–3.492	0.896	−3.341–4.064
Medial	0.323	−1.143–2.383	0.702	−2.603–3.524
Posterior	0.873	−3.021–4.132	0.813	−3.117–3.922

**Table 5:** Results of the binary logistic regression model predicting the influence of hindfoot varus and hindfoot valgus on several variants of ankle impingement in the control group.

	Hindfoot Valgus		Hindfoot Varus	
Impingement	*p*-Value	95% Confidence Interval	*p*-Value	95% Confidence Interval
Anterolateral	0.723	−3.530–3.684	0.701	−4.398–2.447
Anterior	0.904	−4.226–5.254	0.914	−6.128–3.894
Medial	0.854	−3.991–4.295	0.786	−5.386–2.896
Posterior	0.891	−4.195–4.901	0.844	−5.032–3.364

**Table 6:** Results of the binary logistic regression model predicting a potential correlation between articular effusion and hindfoot malalignment or ankle impingement in atraumatic non-overload patients.

	Articular Effusion in the Upper Ankle Joint	
	*p*-Value	95% Confidence Interval
Hindfoot valgus	0.436	−2.182–2.352
Hindfoot varus	0.814	−4.635–5.446
Anterolateral impingement	0.342	−2.008–2.136
Anterior impingement	0.798	−4.454–4.974
Medial impingement	0.717	−3.844–4.639
Posterior impingement	0.736	−4.012–4.822

## Data Availability

The datasets generated during and/or analyzed during the current study are available from the corresponding author upon reasonable request.

## Abbreviations

MRI	Magnetic resonance imaging;
PACS	Picture archiving and communication system;
PD-tse-fs	Fat-saturated proton density-weighted turbo spin echo;
TR	Repetition time;
TE	Echo time;
mm	Millimeter.
